# The effect of concussion history on cognitive-motor integration in elite hockey players

**DOI:** 10.2217/cnc-2016-0006

**Published:** 2016-09-06

**Authors:** Johanna Hurtubise, Diana Gorbet, Yehyah Hamandi, Alison Macpherson, Lauren Sergio

**Affiliations:** 1 School of Kinesiology & Health Science, York University, Toronto, ON, M3J 1P3, Canada; 2 York University Sports Medicine Team, York University Department of Athletics and Recreation, York University, Toronto, ON, M3J 1P3, Canada; 3 Center for Vision Research, York University, Toronto, ON, M3J 1P3, Canada

**Keywords:** adolescent, concussion, eye-hand coordination, mTBI, performance, return-to-play

## Abstract

**Aim:**

To observe the effects of concussion history on cognitive-motor integration in elite-level athletes.

**Methods:**

The study included 102 National Hockey League draft prospects (n = 51 concussion history [CH]; n = 51 no history [NC]). Participants completed two computer-based visuomotor tasks, one involved ‘standard’ visuomotor mapping and one involved ‘nonstandard’ mapping in which vision and action were decoupled.

**Results:**

We observed a significant effect of group on reaction time (CH slower) and accuracy (CH worse), but a group by condition interaction only for reaction time (p < 0.05). There were no other deficits found. We discussed these findings in comparison to our previous work with non-elite athletes.

**Conclusion:**

Previously concussed elite-level athletes may have lingering neurological deficits that are not detected using standard clinical assessments.

An estimated 1.6–3.8 million sports-related concussions, or mild-traumatic brain injuries, occur annually in the USA; however, the true incidence rate is likely higher due to the number of unreported concussions [1–3]. Elite athletes are not an exception to this phenomenon, and the potential long-term health effects of head injury have recently been brought to light through highly publicized litigations within national hockey and football leagues [4]. The Concussion in Sport Group has defined concussion as a pathophysiological injury to the brain caused by biomechanical forces, which leads to clinical alterations but no structural damage [5]. Concussions result in a graded set of clinical symptoms, including physical, cognitive, behavioral and sleep disturbances [1,5]. Furthermore, concussions can lead to cognitive decline and motor deficits [5–7]. Using current clinical neuroimaging protocols, no gross structural damage can be seen after a concussion [5]. However, there are a number of molecular and metabolic events that occur after injury that may lead to changes in brain function [8]. This ‘neurometabolic cascade’ leads to an alteration in ion homeostasis, an increase in free radicals, a decrease in axonal structural integrity and ultimately, disruption of axonal transportation [8].

The length of time for complete recovery from a concussion-induced altered metabolic state is not yet well understood in humans; however, evidence suggests that during the recovery period the brain may be more vulnerable to the effects of a second, or repeated, concussion [9]. In an animal model study by Prins *et al.* [10], it was found that repeated concussions during this period of vulnerability lead to increased axonal injury, memory impairment and risk of mortality. In human studies, it was observed that athletes with a history of three or more previous concussions have a threefold higher risk of sustaining another concussion, and have longer recovery periods [11,12]. Importantly, evidence of increased vulnerability to subsequent concussion during the recovery period indicates that returning an athlete to sport too soon after a concussion may put the athlete at greater risk for further damage, and increase the potential for second impact syndrome and death [1,5,9–10,12]. Therefore, there is a need to better understand the underlying mechanisms and neurological effects of concussion. From a practical standpoint, such an understanding would inform clinicians, charged with making return to play decisions, about the potential for increased vulnerability postconcussion.

Current international protocols recommend that the athletes must be evaluated by a physician and meet baseline measurements of both cognitive and motor assessment tools prior to beginning graded physical activity progressions [3,5]. However, recent studies have shown that commonly used assessment tools, such as neurocognitive testing, and the Sport Concussion Assessment Tool (SCAT3), may not be sensitive enough to determine if the athlete is safe to return to sport [1,2]. These current evaluation tools measure motor and cognitive functions sequentially. In contrast, effective sport performance typically requires their simultaneous involvement – thinking and moving at the same time. To be successful in many sports, a player must apply a wide range of cognitive factors to each of their movements within the game. For example, movements must be made in the context of game-related rules, spatial information regarding the locations of other players and prior knowledge of how to best accomplish a given task. Importantly, this cognition and action is not done sequentially, but rather, concurrently [13,14]. The necessity of this complex cognitive-motor integration (CMI) during game play may help explain why assessment tools that test cognitive and motor functions independently can lack the sensitivity required to detect ongoing concussion-related symptoms.

CMI can be examined in the laboratory by manipulating the mapping of visual information to a required motor response. ‘Standard’ visuomotor mapping involves directly interacting with the object, so that the visual stimulus guiding an action is also the target of the action. A common example of this is looking at and then reaching for a coffee cup, or a pass in hockey when looking at one’s teammate. In contrast, ‘nonstandard’ visuomotor mappings decouple vision and movement, must be learned or calibrated, and require integration of spatial or cognitive rules [15]. A common example of a nonstandard visuomotor mapping in daily life is the use of a computer mouse. To successfully use a computer mouse, one must slide the hand forward on a horizontal plane in order to move the cursor upward on a vertical monitor. In hockey, an example would be passing to one’s teammate on the left, while looking and attempting to avoid a body check from an opponent on one’s right. Previous research has found changes to the movement kinematics of both the eye and hand as a consequence of decoupling vision and movement [16]. Further, the ability to produce decoupled movements is shown to be more vulnerable than the ability to perform standard visuomotor mapping in some forms of clinically altered brain function (Alzheimer’s disease, mild cognitive impairment) [17,18].

With respect to concussion, previous research using nonstandard visually guided arm movements revealed differences in performance of collegiate level athletes, adolescents and children with a previous concussion compared with control participants without a history of concussion [13,14]. Significant group differences were found in reaction time (RT), movement time, and precision, in that those with a previous concussion had performance deficits in both motor planning and execution compared with healthy controls, and this was seen more so in the nonstandard task with decoupled vision and action [13]. These studies all looked at nonelite level athletes. While current protocols suggest that concussion management should be the same for both elite and nonelite athletes [5], some literature suggests that the elite population may be an exception to the standard return-to-play protocol [19]. A study on National Football League (NFL) players, found no significant risk of a subsequent injury when returned to sport on the same day [20], while another study noted that professional athletes performed better in neuropsychological testing post-concussion compared with nonelite athletes [21]. Conversely, it has also been found that retired professional contact-sport athletes with a history of repeated concussion had a fivefold prevalence of being diagnosed with mild cognitive impairment than their peers with no history of concussion [22]. Therefore it is important to understand the effects of concussion in this specialized group, in order to determine effective return-to-play standards. The aim of this study is to better understand how a history of concussion affects CMI in the elite athletic population. We hypothesize that, similar to the collegiate athletes and youth previously studied in our laboratory [13,14], testing with a nonstandard visuomotor mapping that requires CMI will also reveal performance deficits in the young elite-level athlete.

## Methods

### Participants

Participants were selected from the National Hockey League (NHL) draft combine from the 2012–2014 selection years. All athletes invited to the combine, a total of 322 athletes (mean age 17; all identified male), completed the task. Prior to testing, all athletes underwent medical examination with a team of physicians who reviewed the medical history of every prospect player and recorded the athlete’s self-reported history of concussion. A total of 77 athletes reported at least one previous concussion. Athletes who were not medically cleared to participate in sport were excluded from analysis, thus all included participants were medically cleared (including vision), asymptomatic, and currently participating in sport. Data that were missing or unable to be analyzed due to computer malfunction were eliminated from further analysis. Healthy control participants, with no self-reported history of concussion, were randomly matched (randperm function; Matlab R2013b, The Mathworks Inc., Natick, MA, USA) to those with a history of concussion from the same collection year (2012, 2013 or 2014). Participants who were greater than two standard deviations from the mean for any dependent variable were deemed an outlier and removed from further analysis for a total of 102 athletes included in this study; 51 had a self-reported history of concussion (mean age 17 years ± 1) and 51 healthy controls (mean age 17 years ± 1). Information on position, number of previous concussions sustained as well as the length of time (in months) since their last concussion was also obtained during the medical examination (see Tables 1 & 2). All participants completed an informed consent, administered through NHL central scouting, provided to the athletes prior to the day of testing. Ethics were approved through York University (Toronto, ON, Canada).

**Table 1.** **Demographic information by group and position.**

**Position**	**Concussion history; n (%)**	**Healthy control; n (%)**	**Total; n (%)**
Forward	14 (27.5)	16 (31.4)	30 (29.4)

Defense	33 (64.7)	29 (56.9)	62 (60.8)

Goalie	4 (7.8)	6 (11.8)	10 (9.8)

Total	51	51	102

**Table 2.** **Concussion history information.**

	**n (%)**
Previous number of concussions:	
– 1	40 (78.4)
– 2+	10 (19.6)
– Missing	1 (2.0)
– Total	51

Length of time since last concussion:	
– <12 months	17 (33.3)
– 12–24 months	14 (27.5)
– >24 months	15 (29.4)
– Missing	5 (9.8)
– Total	51

### Procedure

Participants completed two computer-based visuomotor transformation tasks, one standard and one nonstandard (vision and action decoupled). Participants sat at a desk so they could comfortably reach a dual-touch-screen 15” laptop (ACER Iconia 6120, Acer America, San Jose, CA, USA) allowing for a screen in both the horizontal and vertical planes. In this way the horizontal screen was well within the comfortable reach range of the participant while the vertical screen was at or slightly below the eye level. In both conditions, participants were instructed to slide their index finger of their dominant hand along the touch screen in order to displace a cursor from a central target to one of four peripheral targets (up, down, left, right) as quickly and as accurately as possible. The central and peripheral targets were viewed on a black background to ensure good contrast. Participants guided a crosshair cursor to the yellow central (or home) target which would change to green when entered. After 4000 ms, a red peripheral target was presented and the central target disappeared, which served as the ‘Go’ signal for participants to initiate movements. Once the cursor reached and remained in the peripheral target for 500 ms, it disappeared, signaling the end of the trial. The next trial began with the presentation of the central target after an intertrial interval of 2000 ms. Peripheral targets were located 75 mm from the center target and target diameters were 20 mm. In order to ensure smooth movement of the finger during the task, participants wore a capacitive-touch glove on their preferred hand.

**Figure 1.**
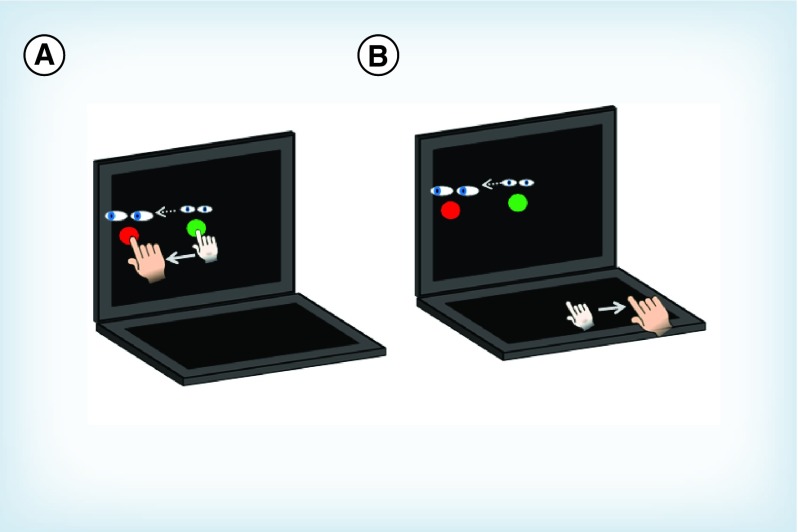
**Illustration of computer-based cognitive-motor integration task.** The green circle denotes the central home target in which all movements begin. A red target appears in one of four peripheral directions (90° to top, bottom, left or right of center) after 4000 ms which serves as the ‘Go’ cue. **(A)** The standard condition, in which eye and arm movements are congruent (moved to the same peripheral target). **(B)** The non-standard condition, in which vision and movement are decoupled due to a plane dissociation (eyes look at vertical screen while hand moves along horizontal screen), and visual feedback reversal (cursor movement 180° rotated from hand motion).

Participants completed the standard and nonstandard conditions in a randomized block design (Figure 1). In the standard condition, participants both looked and moved on the vertical screen, and directly interacted with the targets. The nonstandard condition included two levels of decoupling: plane change, in which participants looked at the vertical screen while moving on the horizontal screen, and cue reversal, in which the feedback was rotated 180° (i.e., in order to move the cursor left, you must slide your finger right). Four trials in each of the four directions were completed per condition for a total of 32 trials per participant (4 directions × 4 trials × 2 conditions). Participants were instructed to move as quickly and accurately as possible, and to look at the targets and not their hands (i.e., no central fixation). Eye movements were monitored by the experimenter to ensure participants were looking at the target; however, a vision tracking system was not used in this experiment. The total time to complete the experiment was typically 4–6 min for each participant. This was a novel task for all participants, with no practice trials allowed; therefore no participant had familiarization of the task. The order of conditions was randomized in order to control for potential learning effects.

### Data processing

Custom-written (C++) acquisition software sampled the finger’s X-Y screen position at 50 Hz. Custom analysis software (Matlab, Mathworks, Inc.) processed individual movement paths with a fourth-order (dual pass) low-pass Butterworth filter at 10 Hz. Filtered paths were then used to generate a computerized velocity profile of each trial’s movement. The movement onsets and ballistic movement offsets (the initial movement prior to path corrections) were scored at 10% peak velocity (PV), while total movement offsets were scored as the final 10% PV point once the finger position plateaued within the peripheral target. Note that in situations where the initial movement successfully brought the finger to the peripheral target, the ballistic and total movement offsets would be equivalent. These profiles were then verified by visual inspection, and corrections were performed when necessary. Trials were deemed errors if the finger/cursor left the center target too early (<4000 ms), RT was less than 150 ms or more than 8000 ms or movement time was more than 10,000 ms. Trials in which the first ballistic movement exited the boundaries of the center target in the wrong direction (>45° from a straight line to target) were coded as direction reversal errors (DR), eliminated from further evaluation and were analyzed as separate variable (see below). The scored data were then processed to compute nine different movement timing and execution outcome measures, described in detail below.

### Movement timing

The measured kinematic variables for movement timing were as follows: RT; the time interval (milliseconds [ms]) between the central target disappearance and movement onset. Movement time; the time between movement onset and offset (ms), calculated as both full movement (TMT, full movement offset) as well as ballistic movement (MT, initial movement offset). If no corrected movements were made, ballistic movements were equal to full movement trajectories. PV; the maximum velocity obtained during the ballistic movement.

### Movement execution

Kinematic variables for movement execution were: Path length; the total distance (resultant of the x and y trajectories) traveled between movement onset and offset (millimeters [mm]), calculated as both the full path length (FPL, full movement offset) as well as the ballistic trajectory (BPL, initial movement offset). Constant error (CE, accuracy); the average distance from the individual ballistic movement end points (∑ x/n, ∑ y/n) to the actual target location (mm). Variable error (VE, precision); was calculated as the distance between the individual ballistic movement end points (σ2) from their mean movement (millimeters; mm). DR errors were calculated as a deviation of greater than ± 45° from the direct line between the center of the central and peripheral targets.

### Performance as a level of dissociation

In order to test the hypothesis that performance declines would be significant for the nonstandard (decoupled) task as compared with the standard task, we compared the athletes’ performance as a function of their change from their own standard condition behavior. We subtracted the result of the standard condition from the nonstandard condition for each of the given dependent measures.

### Data analysis

For eight of the dependent variables described above (RT, MT, TMT, PV, BPL, FPL, CE, VE) main effects of group (concussion history, control) and condition (standard, nonstandard) were analyzed using repeated mixed-model ANOVA. The number of DR, seen only in the nonstandard condition, was analyzed using one-way ANOVA with group (concussion history, control) as the between-subjects factor. To verify significant results, a pairwise comparison was used to test main effects of group for each condition.

Because condition as a factor was significantly different across all variables, we performed further statistical tests using the level of dissociation (delta) in order to compare participants’ performance as a function of their change from the nonstandard condition to the standard condition (see above). One-way ANOVA, comparing group (concussion history, control) across all dependent variables (delta scores; performance) was performed. One-way ANOVA was used to test the effects of the number of previous concussions (1, >2), as well as the length of time since their last concussion (<12 months, 12–24 months, >24 months) on performance. Information as to the number of previous concussions and the length of time since the most recent concussion was not available for all participants, these data were coded as missing and excluded from this analysis. The effect of position (forward, defense, goalie) on performance was analyzed by two-way ANOVA. Correlation of significant variables to both the number of previous concussions and to the length of time since the last concussion was run using point-biserial analysis.

All data were checked for normal distribution (Shapiro–Wilk’s test), homogeneity (Levene’s test; p < 0.01), sphericity (Mauchly’s test) and was Greenhouse–Geisser corrected where necessary. Statistical significance levels were set *a priori* to < 0.05. All statistical analyses were performed using SPSS statistical software (SPSS, IBM Corp. released 2013. IBM SPSS Statistics for Windows, Version 22.0, Armonk, NY, USA).

## Results

**Figure 2.**
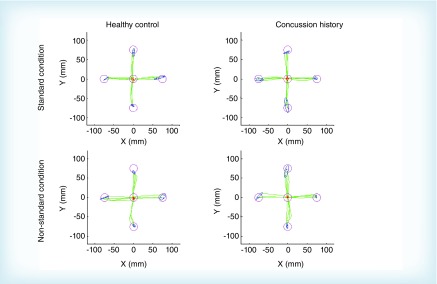
**Fullhand movement trajectories of one healthy control and one concussion history participant in both the standard and non-standard conditions.** Red circle and dots represent the center/home target and initial finger position, pink circles represent the 4 peripheral targets, blue dots represent final end points of individual movements, green lines represent finger trajectory for individual movements, blue ellipses represent the 95% CI surrounding the final end point locations.

Of the total number of athletes tested, 23.9% reported at least one previous concussion. After outlier removal, 102 athletes were included in this study, 51 with a previous concussion and 51 matched healthy controls. Of those who reported a previous concussion, 78.4% (n = 40) reported only one prior injury while 19.6% (n = 10) reported two or more (see Table 2). The length of time since their last concussion was distributed among less than 12 months (n = 17; 16.7%), between 12 and 24 months (n = 14; 13.7%), or greater than 24 months (n = 16; 14.7%) (see Table 2). The majority of the athletes included in the analysis were forward (60.8%, Table 1). No significant relationship between the proportion of concussions sustained and player position was found (χ^2^ [4] = 7.457; p = 0.114). Examples of typical movement trajectories of one subject with concussion history and one healthy control subject performing in both the standard and nonstandard conditions is shown in Figure 2. Descriptive statistics and statistical outcomes of the repeated mixed-model ANOVA for all dependent variables for group (concussion history, control) and condition (standard, nonstandard) are summarized in Table 3.

**Table 3.** **Descriptive statistics of group, by condition, and statistical outcomes of Repeated Mixed-Model ANOVA.**

**Kinematic outcome variables**	**Healthy control; mean (SD)**	**Concussion history; mean (SD)**	**Repeated mixed-model ANOVA statistical outcomes**		

			**Condition**	**Group**	**Group × condition**
**Reaction time (ms)**					

Standard	412.69 (48.840)	425.69 (42.224)	F = 441.410**	F = 6.669^N.S.^	F = 4.630*

Nonstandard	551.26 (73.203)	595.89 (93.176)			

**Ballistic movement time (ms)**					

Standard	264.38 (66.948)	256.85 (65.726)	F = 84.332**	F = 0.362^N.S.^	F = 1.585^N.S.^

Nonstandard	411.11 (204.988)	450.20 (232.864)			

**Total movement time (ms)**					

Standard	298.46 (70.447)	299.69 (70.327)	F = 252.437**	F = 1.856^N.S.^	F = 2.818^N.S.^

Nonstandard	623.36 (251.648)	701.35 (257.510)			

**Ballistic path length (mm)**					

Standard	67.28 (2.960)	66.05 (3.028)	F = 10.973**	F = 2.778^N.S.^	F = 0.108^N.S.^

Nonstandard	65.01 (5.172)	64.19 (5.788)			

**Full path length (mm)**					

Standard	69.85 (2.582)	69.46 (1.987)	F = 43.488**	F = 0.594^N.S.^	F = 1.854^N.S.^

Nonstandard	73.18 (4.682)	74.53 (6.950)			

**Peak velocity**					

Standard	228.07 (51.853)	221.26 (43.531)	F = 157.173**	F = 1.173^N.S.^	F = 0.409^N.S.^

Nonstandard	170.26 (56.853)	157.24 (56.498)			

**Constant error (accuracy) (mm)**					

Standard	7.86 (2.099)	8.71 (2.490)	F = 96.856**	F = 6.340*	F = 0.460^N.S.^

Nonstandard	12.75 (4.630)	14.32 (4.509)			

**Variable error (precision) (mm)**					

Standard	5.62 (2.342)	6.24 (3.311)	F = 8.361**	F = 0.070^N.S.^	F = 1.455^N.S.^

Nonstandard	7.04 (2.971)	7.43 (3.633)			

			One-way ANOVA statistical outcome		

**Direction reversal errors (mean number of trials)**					

Nonstandard	1.10 (1.237)	0.94 (0.947)		F = 0.517^N.S.^	

*p < 0.05.

**p < 0.001.

N.S.: Non-significant; SD: Standard deviation.

### Movement timing

**Figure 3.**
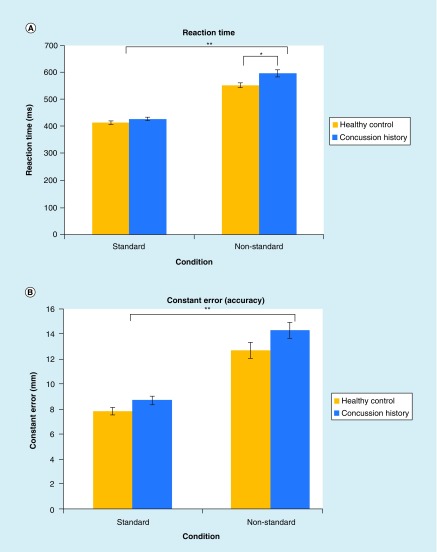
**Significant kinematic variables.** **(A)** Mean reaction time for healthy controls (yellow) and concussion history participants (blue) in both the standard and non-standard condition. Repeated mixed-model ANOVA revealed a statistically significant main effect of condition, group, and a group by condition interaction. **(B)** Mean constant error (accuracy) for healthy controls (yellow) and concussion history participants (blue) in both the standard and non-standard condition. Repeated mixed-model ANOVA revealed a statistically significant effect of condition and group, but not a group by condition interaction (p > 0.05). *p < 0.05. **p < 0.001. Error bars represent SEM.

A repeated mixed-model ANOVA revealed a statistically significant main effect of condition (F[1100] = 441.41; p < 0.001), group (F[1100] = 6.669; p < 0.02) and group by condition interaction (F[1100] = 4.630; p < 0.05) for RT. RT was longer in the nonstandard than in the standard condition, was longer for participants with a concussion history than healthy controls and the interaction revealed that this effect was even more pronounced in the nonstandard condition than in the standard for those with a concussion history (see Figure 3 & Table 3 for values). This finding was confirmed by pairwise analysis; those with a concussion history were significantly slower than healthy controls in the nonstandard (F[1100] = 7.235; p < 0.01), but did not significantly differ in the standard condition (F = 1100) = 2.069; p > 0.05). For MT, TMT and PV, repeated mixed-model ANOVA revealed statistically significant effects for condition, but not for group or group by condition interaction. MT, TMT and PV were significantly longer in the nonstandard condition than in the standard condition, independent of group.

### Movement execution

Repeated mixed-model ANOVA revealed a statistically significant main effect of condition, but not for group or group by condition interaction for BPL (ballistic path length), FPL (full path length) and VE (variable error, precision) (p > 0.05; Table 3). BPL was significantly shorter in the nonstandard condition than in the standard condition; while FPL was significantly longer in the nonstandard condition than in the standard condition, independent of group. VE was significantly larger – denoting decreased precision – in the nonstandard condition than in the standard condition, independent of group. CE revealed a statistically significant main effect of condition (p < 0.001) and group (p < 0.05), but not for group by condition interaction. CE was significantly larger – denoting decreased accuracy – in the nonstandard condition relative to the standard condition, and significantly larger in those with a concussion history compared with no history control participants (Table 3).

DR were observed only in the nonstandard condition. One-way ANOVA revealed no statistically significant differences in the number of errors between those with a history of concussion and healthy controls (F[1100] = 0.517; p > 0.05).

### Performance as a level of disassociation

Descriptive statistics and statistical outcomes of the one-way ANOVA for the level of dissociation on all dependent variables in both groups (concussion history, control) are summarized in Table 4. One-way ANOVA yielded a statistically significant effect of group for RT only (F[1100] = 4.630; p < 0.05); those with a concussion history showing a larger change in performance than healthy controls. All other variables showed no significant differences.

**Table 4.** **Descriptive statistics of group, by level of dissociation (standard - non-standard) and statistical outcomes of one-way ANOVA.**

**Performance score**	**Healthy control; mean (SD)**	**Concussion history; mean (SD)**	
Δ RT	138.58 (68.347)	170.20 (79.652)	F = 4.630*

Δ MT	146.74 (172.747)	193.36 (200.263)	F = 1.585^N.S.^

Δ TMT	324.90 (229.146)	401.66 (232.680)	F = 2.818^N.S.^

Δ BPL	−2.26 (6.326)	−1.85 (6.229)	F = 0.108^N.S.^

Δ FPL	3.34 (5.643)	5.08 (7.157)	F = 1.854^N.S.^

Δ PV	−57.81 (49.806)	−64.03 (48.335)	F = 0.409^N.S.^

Δ CE	4.89 (5.652)	5.61 (5.105)	F = 0.460^N.S.^

Δ VE	1.42 (3.881)	1.18 (5.125)	F = 0.070^N.S.^

*p < 0.05.

BPL: Ballistic path length; CE: Constant error (accuracy); FPL: Full path length; MT: Ballistic movement time; N.S.: Non-significant: PV: Peak velocity; RT: Reaction time; SD: Standard deviation; TMT: Total movement time; VE: Variable error (precision).

Further analysis on performance as a function of the number of previous concussions revealed no statistically significant differences in performance between those with only one previous concussion or those with two or more. The length of time since previous concussion also showed no significant differences in performance. Additionally, a point-biserial correlation found no relationship between performance and the number of concussions previously sustained and no relationship between performance and the length of time since the most recent concussion (p > 0.05). A two-way ANOVA was conducted to examine the effect of position and concussion history on performance; no statistically significant interactions were found across all variables (p > 0.05).

## Discussion

This study sought to determine whether young, elite-level athletes with a history of concussion, who were asymptomatic and medically cleared of concussion, exhibited CMI impairments. A computer-based eye–hand coordination task was used to examine several kinematic variables. Performance deficits in arm RT and constant error (accuracy) of arm movement end points were seen in those with a history of concussion compared with healthy age-, sex- and skill-matched control participants. The deficit in RT was exacerbated in the nonstandard task, where there was dissociation between vision and action requiring rule integration to control goal-directed movements. Although this finding supports our hypothesis of decreased performance with an increase in task complexity in elite athletes, these results differ from our previous findings in collegiate level athletes [14]. Specifically, the collegiate level athletes had deficits in multiple variables including both motor planning (RT), and motor execution (movement time, precision) [14]. While the elite athletes also show an impairment of motor planning (RT) and execution (accuracy), this was seen across fewer variables. Furthermore, while RT was significantly affected by the increase in CMI complexity, ballistic accuracy was impaired independent of condition and therefore these deficits may not be due to a dysfunction within the specific network tested by our task. The performance deficits brought out by testing CMI in this elite sport population were also less severe than those observed in previously concussed select-level youth and adolescent athletes, who displayed an even greater number of impairments in motor planning and execution (movement time, ballistic path length, full path length, accuracy) [13]. Lastly, unlike our previous observations in varsity athletes and select-level youth athletes, the number of previous concussions, the length of time since the most recent concussion or the position played did not influence on any of the variables tested.

Relative to a standard visuomotor mapping, CMI has a greater level of task difficulty due to the simultaneous involvement of cognitive and motor systems, which requires intact reciprocal connections between the frontal (prefrontal, premotor, primary motor), parietal (superior parietal lobule, intraparietal sulcus) and subcortical areas (cerebellum) [23–27]. Task complexity has previously been shown to lead to a decrease in performance, specifically in RT [28], while imaging studies have since shown that the involvement of different brain regions within this frontoparietal network depend on task complexity [24,29–31]. Previous research in our laboratory found an increase in activity in both the inferior parietal lobule and cerebellum during a nonstandard CMI task relative to a standard mapping task. Additionally, spatial patterns of activity within the cuneus and medial premotor cortex were able to differentiate between the two tasks [24]. We propose that the significant differences in performance between our standard and nonstandard task (which were observed in both the concussion and healthy control groups of elite athletes) are due to the increased cognitive load, and thus greater activation within the frontoparietal network for the nonstandard task.

While we observed task-related performance deficits across all participants in the nonstandard task, those with a history of concussion tended to demonstrate even greater decrements relative to healthy controls. Previous research has looked at the effects of a concussion on performance in both standard and simultaneous cognitive motor tasks. A study by Locklin *et al.* [32] found that athletes with a history of concussion had increased mean response times compared with control subjects in a standard mapping reaching task. Although they failed to reach a level of significance, they suggested this may be due to the low difficulty of the task. Additionally, Hugenholtz *et al.* [33] noted that those with a concussion performed significantly slower than controls in choice RT tasks, but not in simple RT tasks; however, the motor aspect of this task was limited to pressing a button, and therefore complex motor kinematic outcomes cannot be inferred. Consistent with these results, we found decreased accuracy in the concussed group across both of our tasks, suggesting that a concussion may impart more general ‘noise’ into the motor system. To our knowledge, our task is the only one which includes a cue reversal as well as a plane change, requiring two levels of vision and motor decoupling, thereby requiring greater recruitment of brain regions within the frontoparietal network responsible for arbitrary mapping and attention. Functional imaging studies have also shown a correlation between frontoparietal network activation and task performance after a concussion [34–37]. Hammeke *et al.* [37] conducted a longitudinal study on National Collegiate Athletic Association (NCAA) football players and found that brain regions associated with attention decreased in activation during the ‘acute’ stage of injury, however, increased in activation during the ‘subacute’ stage, after overt symptoms had resolved. They theorize that during this ‘subacute’ period, neurocognitive functions had improved in order to adequately perform on standardized clinical concussion tests; however, this improvement likely relied on compensatory cognitive mechanisms that resulted in the increased brain activity observed in the ‘subacute’ stage. Further, these findings suggest that performance deficits may appear with an increase in task difficulty that exceeds the capability of the compensatory activity. Similarly, increased brain activity has also been noted within NHL alumni. For example, Esopenko *et al.* [38] found greater activation of the both the prefrontal and posterior parietal regions during a working memory task in those with a history of concussion, despite equivalent behavioral performance when compared with nonconcussed participants. Observations of white matter abnormalities reported in retired NHL players [36], adult female athletes [39] and youth athletes [40] could provide an explanation for observations of increased brain activity post-concussion. We postulate that in the present study, where these athletes’ brains have ostensibly recovered from mild injury, there may still be deficits in the connections between areas required to successfully integrate thought and action [24–25,30,41–44]. That is, we suggest that our observed behavioral deficits may be related to underlying changes in the frontoparietal networks necessary for the successful integration of thought and action, and likely these cortical networks’ interactions with subcortical brain areas, for example, the cerebellum [24,29–30,41–43]. Indeed, we have observed deficits in these brain networks related to impaired performance in this same task in another neurologically compromised group, those at risk for developing dementia [17]. Importantly, on a practical level, our results suggest that athletes with a history of concussion who are slightly slower to react relative to their peers (by ∼10%, in the current study) when simultaneous thinking and skilled action is required may be more vulnerable to sustaining a second concussion during this ‘subacute’ stage. Further, the results presented here and in our previous research suggest that the current return to play assessment – in which thinking and moving are tested separately – does not fully capture the functional disability of a concussion. We postulate that the use of a complex visuomotor task that requires CMI may be more sensitive to the underlying neurological effects of a concussion, and thus may be a useful measure to ensure safe return to sport. While the length of time since their previous concussion was not a significant factor in our study, this may be due to a low sample size within each “time since concussion” subgroup. Previous research in our laboratory, looking at asymptomatic children [14], did find a significant recovery time course. However, further longitudinal investigation on the change in CMI performance in concussion and recovery is needed before its use as a measurement to determine if an athlete is safe to return to play. Importantly, the current task is simple, quick and potentially side-line accessible. Such characteristics are vital since automated devices have recently become the new ‘gold standard’ in concussion assessment since they are simple, portable and reliable [45].

Interestingly, compared with previous findings in varsity-level young adult, nonelite adolescent, and youth athletes [13,14], the results presented here suggest a reduced effect of concussion on CMI in elite athletes. This difference in performance between the elite athletic population and the nonelite athletic populations may be due to a protective effect in the elite brain, a superior CMI frontoparietal network, or potentially a sampling bias (see below). Some speculate that elite level athletes may simply be protected from the effects of a concussion, and thus explains why they are able to compete at the elite level [20,21]. Pellman *et al.* [21] found that NFL athletes had no neurocognitive deficits within the first week after sustaining a concussion, compared with high school athletes who had lingering symptoms. They theorize that this may be due to a higher tolerance in these professional athletes to the effects of concussion. Alternatively, we suggest that this may, instead, be due to the lack of testing a more complex task, one which pushes the movement control system. We propose that elite athletes have a superior frontoparietal network that allows for greater compensation following concussion (i.e., greater motor control ‘reserve’), and thus fewer performance deficits. Studies have shown that elite performers require less neural activation in order to correctly execute a skill, which may be an effect of motor learning [46–48]. Fitts [48] theorized that skilled performers tend to follow patterned processing of skill acquisition, learned through the ‘Associative Stages of Learning’. He postulated that novice athletes must rely on more cognitive processes in order to perform a skill, which is attention-demanding and inefficient, whereas experts have a more automatic performance, which is rapid, smooth and effortless. Granek *et al.* [49], found that expert video gamers displayed a reduction in frontoparietal activity, with increased prefrontal (dorsolateral prefrontal cortex, DLPFC) activity during decoupled (nonstandard) movements compared with nongamers during a CMI task in which the actual performance was equal between groups. Both EEG and fMRI (functional magnetic resonance imaging) studies have shown that expert, or elite, athletes demonstrate ‘neural efficiency’ due to more specific neural circuitry and minimal energy consumption [46,47]. Here we suggest that while behavioral deficits may not be as noticeable in the elite athletic population due to these superior networks, the underlying neural effects may still be present. Further research involving both complex CMI tasks and imaging are needed to better understand the neural effect of mild brain injury on this unique population.

When interpreting the results of this study, it is important to acknowledge that our participants were all NHL draft combine invites at the top of their performance level. These athletes likely played on the highest skilled teams as children. Relative to novice level teams, elite level teams often receive a higher level of medical attention, which may have impacted the care these individuals received after sustaining a concussion and ultimately, their recovery [19]. Therefore, it is difficult to disentangle whether our findings of a potentially increased cognitive-motor reserve in elite athletes is an innate characteristic of these individuals or a product of their environments and training conditions. Additionally, all concussions were self-reported; therefore we cannot be certain that those in our no-history control group had not sustained an unreported concussion. However, as mentioned above, these elite level athletes have likely received higher levels of medical attention throughout their careers and thus likely had better assessment and diagnosis of concussion. Furthermore, we cannot generalize these results to other elite level athletes, since there may be sport-related differences (e.g., in mechanisms of injury), which needs to be further investigated. In addition, while we found no significant effects of position, the number of previous concussions sustained, or the length of time since a participant’s most recent concussion, these results should be interpreted with caution due to the low sample size of some of these subgroups within our main concussion group (see Tables 1 & 2), and potential inaccuracies due to the fact that many concussions likely go unreported. Lastly, while this study did not measure vision or oculomotor control, we acknowledge that it may play an important role in our task, specifically for those suffering an acute concussion. Our participants were asymptomatic and medically cleared by a physician for vision deficits; however, future research should investigate the effects of vision and oculomotor control in CMI tasks, especially in those with an acute concussion. Importantly, for this study we were only able to assess elite male athletes. Given the increasing awareness of the effect of sex (and the completely unstudied effect of gender) on rates of and response to mild head injury [50–52], it will be crucial to repeat this study in elite female athletes in order to adequately address the behavioral effects of concussion history on performance. Future work will utilize imaging in order to investigate the underlying neurological effects of concussion on CMI and its associated networks. Furthermore, a prospective longitudinal study is needed in order to investigate the effects of a concussion on CMI over time. Further research on the specificity, sensitivity, and reliability of this computer-based CMI task is needed to determine its usefulness as an objective and thorough measurement of an athlete’s readiness to return to sport.

## Conclusion

Elite level athletes may have lingering neurological deficits after sustaining a concussion, with impairment in movement planning during complex (nonstandard) visuomotor tasks and in overall accuracy of visually guided reaching movements. Our computer-based CMI task, which requires an arbitrary rule association through decoupling vision and action, is able to identify this deficit. This worsening in RT during tasks which require simultaneous CMI (an important aspect of sport) may leave the athlete more vulnerable to subsequent concussions and their potential long-term effects. These results suggest that the current return to play assessments – in which thinking and moving are tested separately – do not fully capture the functional disability of a concussion and therefore future research focusing on their integration (CMI tasks) is needed.

**Executive summary**

- We investigated whether asymptomatic elite-level adolescent athletes with a history of concussion showed prolonged cognitive-motor integration (CMI) deficits.
- In total, 51 asymptomatic National Hockey League draft prospects with concussion history and 51 controls with no history performed two tasks on a dual-touchscreen laptop. In the one task, target location and motor action aligned, and in the CMI task, target location and motor action were decoupled.
- In the concussion history group we observed significantly slower reaction times during the CMI task, and decreased accuracy in both conditions, relative to controls.
- Based on previous findings, we suggest that these performance deficits are due to concussion-induced disruptions in the fronto-parietal networks responsible for rule-based movement guidance, despite a superior 'motor control reserve' in elite-level athletes.
- The observed CMI deficits suggest that current return to sport assessments that do not test CMI, crucial for skilled activities, are not fully capturing functional abilities post-concussion.
- CMI may provide a more sensitive test even in elite-level athletes prior to return to sport.
